# Comparison of position, morphology and calcification of Coronary Plaque with 320-row dynamic volume CT (DVCT) and coronary angiography (CAG)

**Published:** 2014

**Authors:** Xiaoyan Li, Guoming Zhang, Hongming Zhang

**Affiliations:** 1 Xiaoyan Li, Department of Cardiology, The General Hospital of Jinan Military Region, Jinan 250031, P.R.China.; 2 Guoming Zhang , Department of Cardiology, The General Hospital of Jinan Military Region, Jinan 250031, P.R.China.; 3 Hongming Zhang, Department of Cardiology, The General Hospital of Jinan Military Region, Jinan 250031, P.R.China.

**Keywords:** Calcification, Coronary angiography, Dynamic volume CT, Position, Plaque morphology

## Abstract

***Objective:*** The judgment of coronary stenosis by 320-row dynamic volume CT (DVCT) and coronary angiography (CAG) is not entirely consistent. We compared them in this study.

***Methods:*** We studied 92 patients (265 lesions) with CAG and DVCT. According to the degree of matching in stenosis judgment, all lesions were divided into consistent group and inconsistent group. The position of lesions, the degree of bending, plaque morphology, and calcification proportion were analyzed.

***Results:*** There were 236 lesions in consistent group and 29 lesions in inconsistent group. In inconsistent group, there were more LCX lesions, distal lesions, ostial lesions and tortuous lesions than those in consistent group (*P*<0.05). At the same time, the proportion of nubbly type plaque, mixed plaque, nubbly and nodular type calcification in the main plaque in inconsistent group were higher than those in consistent group (*P*<0.05). The lesions in inconsistent group showed higher calcification load, such as higher segmental coronary calcium score, more calcification points and higher calcification proportion than those in consistent group (*P*<0.05).

***Conclusion:*** When coronary lesion is in ostial, distal or tortuous position, and when the main plaque is nubbly type or with heavy calcification load, the judgment of stenosis by DVCT and CAG is more apt to inconsistent.

## INTRODUCTION

In recent years, with the changes of lifestyle, the incidence of coronary heart disease (CHD) is increasing year by year, which has become one of the most life-threatening diseases. Thus, the "early-discover, early-diagnosis, early-treatment" strategy has become more and more important in the prevention and treatment of CHD. CAG, owing to its highest spatial and temporal resolution ever since, is regarded as the gold standard in the diagnosis of coronary stenosis. Intravascular ultrasound and optical coherence tomography, though with better spatial resolution, is difficult to widely used in clinical practice because they are invasive examinations and expensive. The recent development of MDCT in coronary angiography has made amazing breakthrough in the detection of coronary lesions. From the application of 4-row MDCT in 1998 to the newly-developed 320-row DVCT in 2008, the quantificational assessment of coronary stenosis is becoming more and more precise, which is reckoned as a relatively safe and relatively economic method for early-screening and diagnosis of CHD.^1^^-^^3^

Many studies have shown that coronary CT (CTA) has similar function in diagnosis of coronary stenosis compared with CAG, and has a higher predictive value of the negative lesions,^4^^,^^5^ but it is not completely consistent with myocardial perfusion indicators in evaluating the degree of stenosis. Thus, CTA cannot fully represent the evaluation and examination of patients with medium-to-severe coronary stenosis. Because CTA is noninvasive and low prices, it is widely applied in clinic. So in what lesions the results of CTA and CAG will be different and how to treat the results of CTA in complicated lesions, these are an urgent problem that need to be solved. To get a better understanding of the inconsistency between these two methods and in what condition may this inconsistency occur, we compared 92 patients who underwent both CTA and CAG.

## METHODS

The ethics committee of the General Hospital of Jinan Military Command approved all the study protocols and the informed consents were obtained from all patients or relatives before CTA and CAG.

***General information: ***In this study, patients who visited the Department of Cardiology, General Hospital of Jinan Military Command from January 2010 to May 2012 were selected. The enrolled patients must meet the following criteria: 1. Different degree of coronary stenosis were found through CTA examination (320-row DVCT); 2. CAG examination in the following two weeks confirmed the existence of coronary stenosis. 3. Acute coronary syndrome did not happen one month before and after the CTA and CAG examination.4) CTA and CAG showed good imaging quality; 5) there was no history of the following conditions: allergy to iodine preparation, implantation of a permanent cardiac pacemaker, artificial cardiac valve replacement, severe cardiac or renal insufficiency, and inability to hold breath.

***CTA examination: ***Toshiba Aquiilion one 320-row DVCT (Siemens, Germany) was used for CTA. Scanned area was as follows: from the point below carina to about 1 cm below diaphragmatic muscle. Sure Start software was used for intelligent trigger scan. The trigger point was thoracic aorta on the central plane of the scanned area. Trigger threshold was 180 Hu. Scan parameters were as follows: 120 kV, 500 mA; the area of volume data collection was 320 rows ×0.5 mm; rotation speed of the gantry was 350 ms. Scan time was about 0.35-1.4 s. Clear data obtained from reconstruction were transferred to Vitrea II fx specific graphic processing workstation, and post-processing was performed with corresponding software systems. The following indicators were analyzed by two experienced doctors in CT room. If there was disagreement, re-evaluation was performed till consensus.  

Degree of coronary stenosis: visual measurement of diameter which was commonly used internationally was adopted, i.e. degree of vascular stenosis = (normal vascular diameter at proximal part of stenosis – diameter of stenosis)/normal vascular diameter at proximal part of stenosis × 100%.

Morphology of main plaque and calcification: the site with the most severe luminal stenosis was analyzed for each coronary segment. In case of coronary occlusion, the segment distal to occlusion was not analyzed. Long-axis image obtained from curved planar reformation was used to analyze coronary morphology at the long-axis direction. Then the curved planar reformation image was rotated to locate the plaque at the tangential position of the lumen, so as to observe morphology of the plaque (calcification) at the position, and perform classification analysis of coronary plaque (calcification). Morphology of the plaques causing the most severe stenosis was analyzed in long-axis image obtained from curved planar reformation, and the plaques were classified according to the method of Kajinam^   4 ^. Classification criteria were as follows: 1) nubbly type: length of the plaque > 2/3 inner diameter of the reference blood vessel, and width of the plaque > 2/3 inner diameter of the reference blood vessel; 2) nodular type: length of the plaque < 2/3 inner diameter of the reference blood vessel, and width of the plaque > 2/3 inner diameter of the reference blood vessel; 3) strip type: length of the plaque > 2/3 inner diameter of the reference blood vessel, and width of the plaque < 2/3 inner diameter of the reference blood vessel; 4) focal type: length of the plaque > 2/3 inner diameter of the reference blood vessel, and width of the plaque < 2/3 inner diameter of the reference blood vessel. For classification of morphology of calcification in the plaque was compared with width of the plaque.  

Proportion of calcification in the main plaque: for the main plaque causing the most severe stenosis, curved planar reformation image was taken. Image pro plus 5.02 was used to outline calcification spot and plaque areas and calculate the proportion.

Determination of segmental coronary calcium score (SCCS): according to Agastton method^   5 ^, if calcification area was more than 1 mm2, and peak factor of CT value was more than +130 HU, it was regarded as a calcification lesion. Calculation of SCCS for each calcification lesion was as follows: peak factor of CT value × corresponding calcification area (unit: mm2).

***CAG examination: ***Within two weeks of CACS determination, all the selected 96 patients underwent CAG. Artis dTA (Siemens, Germany) was used. Two experienced cardiologists evaluated the degree of stenosis for all coronary segments with luminal diameter =1.5mm, using visual measurement of diameter which was commonly used internationally (percentage of the difference between vascular diameter of the lesion and vascular diameter proximal to the heart in vascular diameter proximal to the heart). If there was disagreement, re-evaluation was performed till consensus.

***Study protocols: ***The most severe lesion was analyzed for each segment of coronary artery. According to the body position where CAG visualized coronary stenosis best, CTA image was simulated and reconstructed. Corresponding position was found in the image, and degree of coronary stenosis at the position was recorded, which was luminal stenosis in CTA. Degrees of stenosis in CAG and CTA were both divided into four intervals: 0-30%, 30-50%, 50-75% and above 75%. Lesions evaluated with the two methods were compared in each segment. Stenosis evaluations in the same interval were included in consistent group, and evaluations in different intervals were included in inconsistent group.

***Statistical analysis: ***CAG results were used as the gold standard. SPSS 17.0 software was used for statistical analysis. Quantitative data were expressed as mean ± standard deviation. In comparison of means from the two samples, independent sample t test was performed. For qualitative data, X^2^ test was used. In all analyses, P<0.05 was considered significant.

## RESULTS

***General information: ***Ninety two patients which included 59 men and 33 women were enrolled, The mean age was 60.81±9.52 years. There were totally 265 lesions.

***Lesion positions: ***Segmentation of lesions was performed according to ACC coronary 15-segment method. The results showed no significant difference for each segment. However, after re-combination, there were much more lesions located in LCX, distal and ostial position in inconsistent group than that in consistent group (Table-I, p<*,*0.05).

***Lesion morphology and characters: ***Morphology and characters of the main plaque were classified according to various classification methods. The results showed that proportions of lesion with nubbly type plaque and tortuous lesion in inconsistent group were significantly higher than consistent group (Table-II, p*<*0.05).

***Lesion calcification: ***As shown in Table-III, in inconsistent group, proportion of mixed plaque was higher, segmental calcification score was higher, number of calcification in the main plaques was higher, and there were mostly nubbly and nodular types in the main plaques. The above differences all had statistically significant level (*P <*0.05).

## DISCUSSION

In diagnosis of coronary heart disease, coronary angiography is the most common method, as well as the “gold standard”. However, as it is invasive examination, it still requires inpatient treatment in most regions in China. CTA is simple, rapid and convenient; it can reduce unnecessary interventional diagnosis, as well as economic cost and suffering of patients. With rapid development of multi-layer spiral CT in recent years, 16-layer CT, 64-layer CT, dual-source CT and 320-row dynamic volume CT (DVCT) emerge successively, and are gradually applied in coronary angiography.^6^^-^^8^ However, due to various factors, evaluation of coronary stenosis with CTA is not as accurate as coronary angiography. Previous studies indicate that CTA has a good negative predictive value but poor accuracy.^3^^,^^9^^,^^10^ Therefore, investigating relevant factors causing inaccurate stenosis evaluation with CTA will providing assistance in more accurate interpretation of CTA images, and more accurate reference information in clinical diagnosis and treatment.

**Fig.1:**
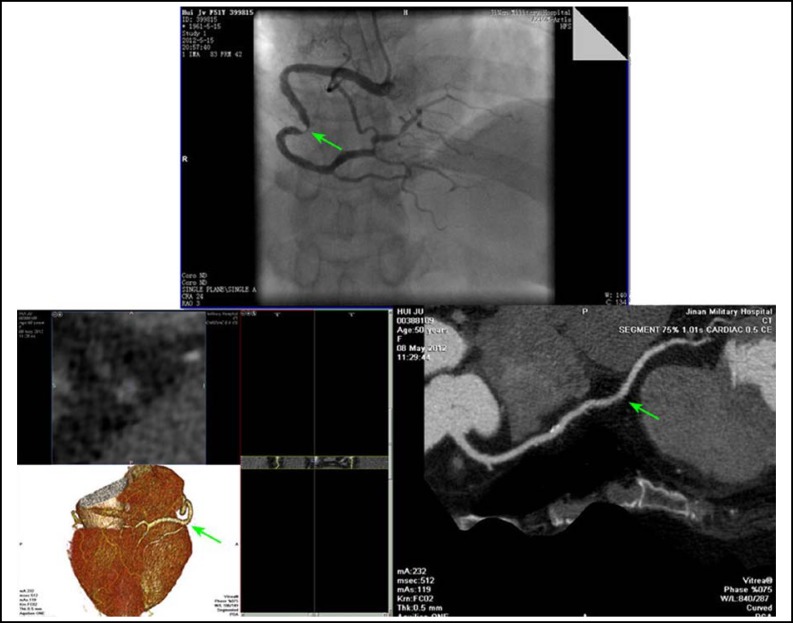
Tortuous lesion effected judgment of coronary stenosis degree by CAG and CTA. CAG showed severe stenosis in the middle of RCA, CTA showed no obvious stenosis

**Fig.2:**
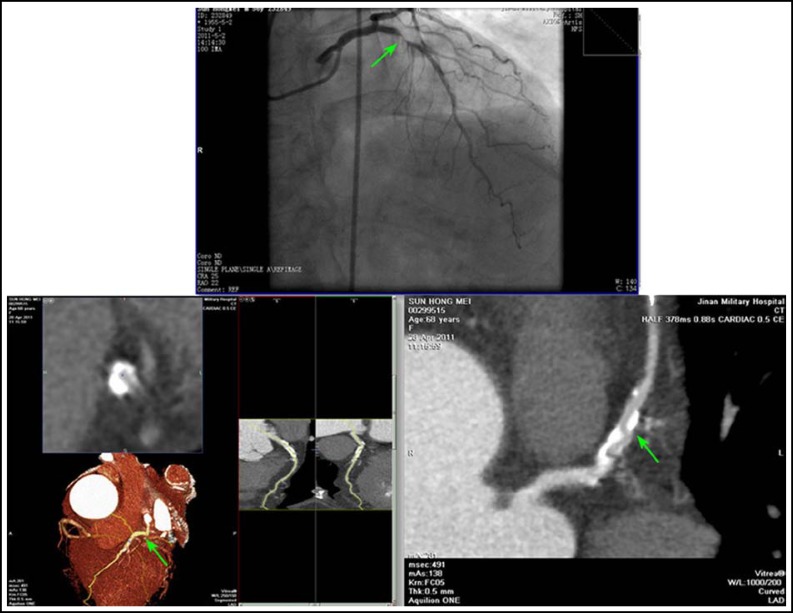
Severe calcification effected judgment of coronary stenosis degree by CTA. CAG showed severe stenosis of 99 percent in the proximal of LAD, CTA showed the stenosis of about 30%.

**Table-I:** Comparison of lesion positions

	***Consistent group***	***Inconsistent group***	***P value***
LAD lesion	116	9	0.09
LCX lesion	55	13	*0.02*
RCA lesion	65	7	0.86
Proximal lesion	88	8	0.30
Mid portion lesion	106	8	0.11
Distal lesion	42	13	*0.01*
Ostial lesion	20	8	*0.01*
Non-Ostial lesion	216	21	*0.01*

**Table-II:** Comparison of morphology and characters

	***Consistent group***	***Inconsistent group***	***P value***
Morphology			
nubbly type	64	13	*0.04*
nodular type	55	6	0.75
strip type	79	6	0.69
focal type	38	4	0.74
Character			
diffuse lesion	40	5	0.83
tubular lesion	153	17	0.79
local lesion	43	7	0.86
Tortuous lesion			
yes	11	5	*0.02*
no	225	24	*0.02*
Total occlusion lesion			
yes	6	2	0.47
no	230	27	0.47

**Table-III:** Comparison of calcification in plagues

	***Consistent group***	***Inconsistent group***	***P value***
Character of major plaque			
soft	85	7	0.16
mixed	124	14	0.66
calcific	27	8	*0.03*
SCCS	81.87±66.35	249.89±210.04	*0.01*
Count of calcification	1.29±0.97	1.84±1.06	*0.01*
Proportion of calcification	43.16±29.59	55.33±27.69	*0.02*
Shape of calcification type			
nubbly and nodular	37	10	*0.02*
Strip and focal	114	12	0.48
Non-calcific	85	7	0.28

This study indicated that lesion position and characters were important factors causing inconsistency between CTA and CAG evaluations. There were significant differences in LCX lesion, especially middle lesion of LCX, and tortuous and ostial lesions at other sites between the two groups. This may be because some information was distorted in volume reconstruction for tortuous and ostial lesions in CTA; in addition, blood vessels should be sufficiently unfolded for evaluation of the above lesions in CAG, and routine body position may influence judgment of the results. There was also statistically significant difference in distal lesion between the two groups. The reason may be that CTA imaging is different from CAG dynamic imaging, so contrast filling time in the distal segment may be influenced by stenosis of proximal and middle segments, causing difference in time, and thus influencing routine CTA judgment. Morphology of main plaque is one of the important factors influencing judgment of stenosis degree.^11^^-^^14^ This study indicated that proportion of nubbly plaque in inconsistent group was significantly higher than that in consistent group. This may be because that both length and inner diameter of nubbly plaque are larger than 2/3 inner diameter of the reference blood vessel, so this type of lesion has heavier plaque load than nodular, strip and focal types, and more easily influence evaluation of inner diameter of proximal and distal reference blood vessels, thus resulting in greater difference between CTA and CAG.^4^

Judgment of calcification in coronary lesion is the advantage of CTA. However, partial volume effect caused by high-density plaque influences luminal visualization, and thus influences judgment of coronary stenosis degree.^15^^,^^16^ Results of this study are consistent with the above speculation, indicating number of calcified plaques in inconsistent group was significantly higher than that in consistent group. Moreover, SCCS and proportion of calcification in main plaque in inconsistent group were significantly higher than those in consistent group. Previous studies rarely investigated influence of calcification morphology in main plaque on CTA interpretation. This study showed that proportion of nubbly and nodular type calculation in inconsistent group was higher than that in consistent group. This may be because that larger cross section of calcification causes higher “barrier effect”, increasing the probability of inconsistent evaluation.

This study showed that CTA and CAG had a high consistency rate (78%) in judgment of coronary stenosis degree, and found that inconsistency in judgment of coronary stenosis between the two methods was mainly due to the above reasons. In addition, previous studies indicate that in some total occlusive lesions, good lateral branches may cause filling of contrast distal to the occlusion, or CTA reviewer wrongly judges branch near occlusion as the main trunk, causing missed diagnosis in CTA. In this study, there were relatively few complete occlusive lesions (2%). Maybe due to small sample size, the above results did not show significant difference. Besides, in this study, consistent group was defined as stenosis evaluations with CTA and CAG were in the same interval. Though this design will not influence selection of treatment strategy, in judgment of stenosis degree, this may reduce case number in inconsistent group. This is also the shortcoming of this study. 

In summary, this study indicate that there may be great differences between CTA and CAG in judgment of stenosis degree in respect of ostial, distal or tortuous lesions, as well as in case of nubbly type of plaque, and plaque with high loads of calcification. Therefore, in clinical diagnosis and selection of treatment strategy, in case of the above situations, CTA or CAG results should be carefully checked, so as to avoid misdiagnosis caused by wrong judgment.
